# Mutation of *GmAITR* Genes by CRISPR/Cas9 Genome Editing Results in Enhanced Salinity Stress Tolerance in Soybean

**DOI:** 10.3389/fpls.2021.779598

**Published:** 2021-11-26

**Authors:** Tianya Wang, Hongwei Xun, Wei Wang, Xiaoyang Ding, Hainan Tian, Saddam Hussain, Qianli Dong, Yingying Li, Yuxin Cheng, Chen Wang, Rao Lin, Guimin Li, Xueyan Qian, Jinsong Pang, Xianzhong Feng, Yingshan Dong, Bao Liu, Shucai Wang

**Affiliations:** ^1^Key Laboratory of Molecular Epigenetics of MOE, Northeast Normal University, Changchun, China; ^2^National Engineering Research Center for Soybean, Soybean Research Institute, Jilin Academy of Agricultural Sciences, Changchun, China; ^3^Laboratory of Plant Molecular Genetics and Crop Gene Editing, School of Life Sciences, Linyi University, Linyi, China; ^4^Key Laboratory of Soybean Molecular Design Breeding, Northeast Institute of Geography and Agroecology, Chinese Academy of Sciences, Changchun, China

**Keywords:** *GmAITRs*, salinity tolerance, ABA, CRISPR/Cas9, genome editing, soybean

## Abstract

Breeding of stress-tolerant plants is able to improve crop yield under stress conditions, whereas CRISPR/Cas9 genome editing has been shown to be an efficient way for molecular breeding to improve agronomic traits including stress tolerance in crops. However, genes can be targeted for genome editing to enhance crop abiotic stress tolerance remained largely unidentified. We have previously identified abscisic acid (ABA)-induced transcription repressors (*AITRs*) as a novel family of transcription factors that are involved in the regulation of ABA signaling, and we found that knockout of the entire family of *AITR* genes in Arabidopsis enhanced drought and salinity tolerance without fitness costs. Considering that AITRs are conserved in angiosperms, *AITRs* in crops may be targeted for genome editing to improve abiotic stress tolerance. We report here that mutation of *GmAITR* genes by CRISPR/Cas9 genome editing leads to enhanced salinity tolerance in soybean. By using quantitative RT-PCR analysis, we found that the expression levels of *GmAITRs* were increased in response to ABA and salt treatments. Transfection assays in soybean protoplasts show that GmAITRs are nucleus proteins, and have transcriptional repression activities. By using CRISPR/Cas9 to target the six *GmAITRs* simultaneously, we successfully generated Cas9-free *gmaitr36* double and *gmaitr23456* quintuple mutants. We found that ABA sensitivity in these mutants was increased. Consistent with this, ABA responses of some ABA signaling key regulator genes in the *gmaitr* mutants were altered. In both seed germination and seedling growth assays, the *gmaitr* mutants showed enhanced salt tolerance. Most importantly, enhanced salinity tolerance in the mutant plants was also observed in the field experiments. These results suggest that mutation of *GmAITR* genes by CRISPR/Cas9 is an efficient way to improve salinity tolerance in soybean.

## Introduction

As the fourth major crop and a nitrogen-fixing plant, soybean (*Glycine max*) is one of the most important protein- and oil-rich seed crops worldwide (Zhang et al., 2015; Vanlliyodan et al., 2017), and it plays an important role in maintaining the cycling of nitrogen in ecosystems (Deshmukh et al., 2014). However, similar to other crops, growth and yield of soybean is largely affected by abiotic stresses including drought, salinity and extreme temperatures. As an example, drought alone can cause up to 40% yield loss of soybean globally (Wang et al., 2003; Fujita et al., 2006; Manavalan et al., 2009; Ray et al., 2013). In addition, drought and salinity are common in many different regions, and long-term drought caused by accelerated climate changes and global warming usually led to salinity. As a result, more than 50% of all arable lands on the earth may get seriously salinized by the year 2050, a dramatically increase from a currently ∼20% (Wang et al., 2003). Considering that the world population is continuing increasing and an estimated increase of 70% in crop yield is needed to feed the population by the year 2050 (Ray et al., 2013; Vanlliyodan et al., 2017), crop breeding to enhance abiotic stress tolerance is a critical way to improve crop yield. However, traditional breeding to improve abiotic stress tolerance may take years to decades (Manavalan et al., 2009).

Molecular breeding is able to shorten the time required for crop breeding, and the outcomes are usually more predictable compared to traditional breeding (Xu et al., 2012). The application of new developed techniques such as CRISPR (clustered regularly interspaced short palindromic repeats)/Cas9 (CRISPR -associated protein 9) genome editing in molecular breeding may further shorten the time required for crop breeding (Chen et al., 2019; Matres et al., 2021), as CRISPR/Cas9 genome editing not only enables to generate predictable mutations, but also enables to isolate transgene-free mutants from the edited transgenic plants (Ma et al., 2015; Gao et al., 2016; Lu et al., 2017; He et al., 2018; Chen et al., 2019). Since its successful application in plants (Li et al., 2013; Nekrasov et al., 2013; Shan et al., 2013), CRISPR/Cas9 genome editing has been used to improve agronomic traits in crops such as rice, tomato and wheat by editing specific target genes (Shimatani et al., 2017; He et al., 2018; Zsögön et al., 2018; Chen et al., 2019). However, identification of appropriate target genes that can be used to improve abiotic stress tolerance in crops by CRISPR/Cas9 genome editing is a challenge.

It is well known that ABA (abscisic acid) is a key stress hormone, through the PYR1/PYL/RCAR (Pyrabactin resistance 1/PYR1-like/Regulatory component of ABA receptor) receptors, the A-group PP2Cs (PROTEIN PHOSPHATASE 2C) phosphatases, the SnRKs [NON-FERMENTING 1 (SNF1)-RELATED PROTEIN KINASES] kinases, and the ABF/AREB/ABI5-type bZIP (basic region leucine zipper) transcription factors, ABA regulates the expression of ABA responsive genes and thereby plant responses to abiotic stresses such drought, salinity, cold, and heat (Rodriguez et al., 1998; Gosti et al., 1999; Fujii et al., 2007; Fujii and Zhu, 2009; Umezawa et al., 2010; Guo et al., 2011; Rushton et al., 2012; Yoshida et al., 2014; Dong et al., 2015; Tian et al., 2017). As a result, expression level changes of the ABA signaling regulator genes usually led to changes in plant tolerance to abiotic stresses, but in most of the cases, enhanced abiotic stress tolerance was observed in plants overexpressing the regulator genes, whereas loss-of-function of the regulator genes led to reduced abiotic stress tolerance in plants (Fujita et al., 2009; Park et al., 2015; Yoshida et al., 2015; Zhao et al., 2016). Therefore, it is unlikely for the ABA signaling key regulator genes to be served as target for CRISPR/Cas9 genome editing to improve abiotic stress tolerance in crops.

We have previously identified AITRs (ABA-induced transcription repressors) as a novel family of transcription factors that function as feedback regulators of ABA signaling, and loss-of-function of *AITR* genes led to reduced ABA sensitivity in Arabidopsis (Tian et al., 2017). Consistent with the functions of AITRs in regulating ABA signaling, expression level changes of the *AITR* genes in Arabidopsis also led to changes in plant tolerance to abiotic stresses (Tian et al., 2017; Chen et al., 2021). However, different from most of the ABA signaling key regulator genes, loss-of-function of *AITR* genes resulted in enhanced tolerance to abiotic stresses including drought and salinity, whereas overexpression of *AITR5* led to reduced tolerance to salt stress in Arabidopsis (Song et al., 2016; Tian et al., 2017; Chen et al., 2021). Most importantly, knock-out-of all the six *AITR* genes in Arabidopsis led to enhanced tolerance to drought and salinity without fitness cost (Chen et al., 2021). AITRs are conserved in angiosperms, and our preliminary studies have shown that AITRs from soybean, tomato, rice and cotton shared similar features of the Arabidopsis AITRs, i.e., they are all function as transcription repressors as examined in transfected Arabidopsis protoplasts, and their expression was induced by ABA treatment (Tian et al., 2017; Wang et al., 2021). In addition, expression of a cotton *AITR* gene recovered the abiotic stress tolerance phenotypes observed in the Arabidopsis *aitr2* mutant (Wang et al., 2021), indicating that crop AITRs may have similar functions as Arabidopsis AITRs. These results suggest that *AITRs* may serve as CRISPR/Cas9 genome editing targets to improve abiotic stress tolerance in crops.

We report here the characterization of soybean AITRs (GmAITRs). We found that expression of *GmAITRs* is induced by both ABA and salt, and GmAITRs function as transcription repressors in transfected soybean protoplasts. We generated transgene-free *gmaitr* mutants by using CRISPR/Cas9 genome editing to target *GmAITR* genes, and found that the *gmaitr* mutants showed enhanced tolerance to salt in both laboratory and field assessments.

## Materials and Methods

### Plant Materials and Growth Conditions

Williams 82 (Wm82) wild type soybean (*Glycine max*) was used for plant transformation, protoplasts isolation and as control for the experiments. The transgene-free *gmaitr36* double and *gmaitr23456* quintuple mutants were generated by using CRISPR/Cas9 gene editing in the Wm82 wild type background.

For generation assays, ABA and salt tolerance assays, and gene expression in response to ABA and salt, seeds of the Wm82 wild type and the *gmaitr* mutants were generated on the surface of two layers of wet filter papers in Petri plates or in plastic growth bags (PhytoTC, Beijing) (Li et al., 2019), and grown in a growth room. For gene expression pattern assays and protoplast isolation, seeds were germinated in soil pots and grown in a growth room. For gene expression in response to ABA and salt, or ABA signaling key regulator gene expression, seeds were germinated and grown hydroponically in distilled water. The conditions at the growth room were set at 25°C, with 16 h light/8 h dark light cycle with light density at ∼600 μmol m^–2^ s^–1^, and a 60% relative humidity.

For field production analysis, seeds of the Wm82 wild type and the *gmaitr* mutants were sow and grown in three experimental fields in Jilin province, including two fields with normal soil and one saline-alkali soil field, i.e., normal soil field 1 (E124°48′, N43°30′), normal soil field 2 (E125°05′, N43°44′), and the saline-alkali soil field (E122°45′, N45°20′), in the year 2020. The saline-alkali soil field is a typical saline-alkali land with pH 8.1–9.8, and total soluble salt 0.1–0.7%.

### Sequence Alignment, Conserved Motif Analysis, and Three-Dimensional Protein Structure Prediction of *GmAITRs*

The full-length amino acid sequences of the six GmAITRs identified previously (Tian et al., 2017), were subjected to amino acid sequence alignment by using BioEdit with default settings, to motif analysis by using MEME^1^ with default settings (Bailey et al., 2009), The GmAITR sequences in the Wm82 wild type and the *gmaitr* mutants were used for three-dimensional protein structures prediction by using Alphafold v2.0^2^ with default settings (Jumper et al., 2021). The protein structural alignment and root mean square deviation (RMSD) values were analyzed by PyMOL (The PyMOL Molecular Graphics System, Version 2.0 Schrödinger, LLC.). The protein structure of GmAITR2 was drawn by BIOVIA Discovery Studio Visualizer 2020.^3^

### Phylogenetic Analysis

The full-length amino acid sequences of *GmAITRs*, or *GmAITRs* and AITRs from Arabidopsis, Medicago, and rice were used for alignment on MAFFT^4^ (Katoh and Standley, 2013). Phylogenetic tree was generated based on the sequence alignment result, by using MEGA7 (Kumar et al., 2016). The cross-species analysis of AITRs was performed by using the Neighbor-Joining method based on the Poisson correction substitution model. All ambiguous positions were removed for each sequence pair. The sequences used in phylogenetic analysis have been listed by Tian et al. (2017).

### Abscisic Acid and NaCl Treatment

To examine the expression of *GmAITRs* in response to ABA and NaCl, healthy and uniform-sized seeds of the Wm82 wild type were selected and grown hydroponically in distilled water for 14 days. The seedlings were then transferred to 100 μM ABA, 200 mM NaCl or distilled water as a control, and treated for 6 h. Roots and leaves were dissected from the seedlings immediately after the treatments, frozen in liquid nitrogen and stored in −80°C for RNA extraction.

To examine ABA response of the ABA signaling key regulator genes, seeds of the Wm82 wild type and the *gmaitr* mutants grown in the plastic bags with distilled water for 14 days, then transferred to 100 μM ABA and distilled water as a control, and treated for 6 h. After the treatments, roots were collected and frozen in liquid nitrogen and stored in -80°C for RNA extraction.

### RNA Isolation, cDNA Synthesis and qRT-PCR

For ABA response of *GmAITR* genes and ABA signaling key regulator genes, the above mentioned samples collected were used for RNA isolation. For tissue expression analysis, roots, stems and leaves were collected from 28-day-old soil pot-grown Wm82 wild type plants when the trifoliate leaf fully opened, frozen in liquid nitrogen and stored in -80°C for RNA extraction.

Total RNA was isolated from the samples collected by using an OminiPlant RNA kit (CWBIO) according to the manufacturer’s instructions. During the isolation, RNA was treated with RNase-Free DNase (CWBIO) to avoid the contamination of DNA. After the DNase treatment, 1 or 2 μg total RNA was used to synthesize cDNA by oligo(dT)20-primed reverse transcription using the EasyScript One-Step gDNA Removal and cDNA Synthesis SuperMix (TransGen Biotech). The synthesized cDNA was used as the template for gene expression analysis. For qRT-PCR, each sample was amplified in three parallel reactions as technical replicates, and the *GmEF-1*α (*Glyma.17G186600*) was amplified as a reference gene. The primers used for genes *GmPYL9*, *GmPYL10*, *GmPYL12*, and *GmPP2C1* have been described previously (Bai et al., 2013), and the primers used for expression analysis of other genes are listed in Supplementary Table 1.

### Constructs

The reporter construct *LexA-Gal4:GUS*, and the effector constructs *GD*, *GD-GmAITRs*, *GFP-GmAITRs*, and *LD-VP* have been described previously (Tiwari et al., 2004; Wang et al., 2005; Tian et al., 2017).

To generate CRISPR/Cas9 constructs for *GmAITRs* gene editing, the potential target sequences within the exons of *GmAITRs* were selected by using targetDesign on CRISPR-GE.^5^ Target specificity was then evaluated by using offTarget on CRISPR-GE. A total of six target sequences were selected. Due to the high CDS sequence similarity (>85%) between *GmAITR* gene pairs, i.e., *GmAITR1* and *GmAITR4*, *GmAITR2* and *GmAITR5*, and *GmAITR3* and *GmAITR6*, each of the six target sequences was able to target one pair of genes. The six targets were divided into two groups with each group contains three target sequences that can target all the six genes. The target sequences were inserted into the *pYL-CRISPR/Cas9P_*ubi*_-B* vector to generate CRISPR/Cas9 genome editing constructs using the method described previously (Ma et al., 2015). The target sequences in construct one are 5′-GGATGCACCGGGTACATACC(TGG)-3′ targets *GmAITR2* and *GmAITR5*, 5′-GGAGGGGTTTGGGGGCGATA(GGG)-3′ targets *GmAITR1* and *GmAITR4*, and 5′-GCGTGACAGGCACG TGCATG(GGG)-3′ targets *GmAITR3* and *GmAITR6*. The target sequences in construct two are 5′-GTGGTGTTCGT GTGTGACGG(TGG)-3′ targets *GmAITR2* and *GmAITR5*, 5′-G AGGTTTCACGTGCAGGGTG(AGG)-3′ targets *GmAITR1* and *GmAITR4*, and 5′-GTGAAAGCTGCGCTCAGTTT (GGG)-3′ targets *GmAITR3* and *GmAITR6*. The primers used for making the constructs are listed in Supplementary Table 2.

### Plant Transformation, Transgenic Plant Selection, and Transgene-Free Mutant Isolation

*pYL-CRISPR/Cas9P_*ubi*_-B* constructs for *GmAITRs* were transformed into the *Agrobacterium tumefaciens* strain of EHA105, and then used to transform soybean by using Agrobacterium-mediated cotyledonary node transformation method as previously described (Paz et al., 2004).

Transgenic plants generated were initially examined by using GMO DETECT kit (bar/pat) (Artron Laboratory Inc., Beijing) flowing the manufacturer’s instructions, and then examined by PCR amplification of *Cas9* gene fragment. Gene editing status in the confirmed T1 transgenic plants was examined by amplifying and sequencing the genomic sequence of *GmAITR* genes. Transgene-free homozygous mutants were isolated from T2 progeny of gene edited T1 plants by PCR amplification of *Cas9* gene fragment, and sequencing of *GmAITR* genes.

### DNA Isolation and PCR

DNA was isolated from leaves of the T1 transgenic plants and T2 progeny of gene edited T1 plants by using a method described previously (Edwards et al., 1991).

To confirm the transgenic status of the T1 plants and to isolate transgene-free mutants in T2 progeny of gene edited T1 plants, DNA isolated was used as templates to amplify *Cas9* gene fragment by PCR. The primers used are 5′-CGCTCAGATTGGAGATCAGT-3′, and 5′-CGAAGTT CCAAGGGGTGATA-3′.

To examine genome editing status of *GmAITR* genes, DNA isolated was used as templates to amply genome sequence of *GmAITR* genes by PCR, and the PCR products was isolated and sequenced. The sequencing results were aligned with wild type sequences of the Corresponding *GmAITR* gene. The primers used for PCR amplification of *GmAITR* genes are listed in Supplementary Table 3.

### Plasmid Isolation, Protoplast Isolation, and Transient Transfection

Plasmids of the reporter and effector constructs were extracted using a GoldHi EndoFree Plasmid Maxi Kit (CWBIO) according to the manufacturer’s instructions. Protoplasts were isolated and transfected by following a procedure previously described (Xiong et al., 2019). Briefly, protoplasts were isolated from trifoliate leaves of 2-week-old soil pot-grown Wm82 wild-type plants, plasmids were transfected or co-transfected into the protoplasts isolated, and transfected protoplasts were incubated under darkness at room temperature. For subcellular localization assays, the transfected protoplasts were incubated for 16–18 h, and then GFP fluorescence was examined under an Olympus BX61 fluorescence microscope. For transcription activity assays, the transfected protoplasts were incubated for 22–24 h, and then GUS activities were measured by using a Synergy^TM^ HT fluorescence microplate reader (BioTEK).

### Seed Germination Assays

Healthy and uniform-sized seeds of the Wm82 wild type and the *gmaitr* mutant plants were placed in Petri plates on the surface of two layers of filter papers soaked with 100 μM ABA 200 mM NaCl, or distilled water as a control. The plates were kept in a growth room, and germinated seeds were count at indicated time points. Each plate contains ten seeds and seeds with radicles longer than 0.5 cm were calculated as germinated seeds at the eleven time points (Kan et al., 2015).

### Seedling Growth Assays

Healthy and uniform-sized seeds of the Wm82 wild type and the *gmaitr* mutant plants were germinated and grown with distilled water in plastic growth bags (PhytoTC, Beijing) (Li et al., 2019) for 3 days, and then initiated the salt treatment by adding the 200 mM NaCl solution or fresh distilled water as a control. Two parallel bags were used for each treatment, and two plants for each genotype were included in one bag, and different genotypes in different growth bags were placed in different order to minimize the position effects. After grown in a growth room for 2 weeks, seedlings were taken out from the growth bags for measurement of the shoot and root length.

### Field Production Assays

For the agronomic traits comparison, seeds of the Wm82 wild type and the *gmaitr* mutant plants were sown in the experimental fields in plots by genotypes. Each plot in the two normal soil fields includes four rows, and each plot in the saline-alkali field includes three rows. The plot length was 2 m, the space between rows was 0.5 m, and the space between plants in the rows was 10 cm. The seeds were sown in May and the plants were harvested in October in the year 2020.

### Statistical Analysis

A statistical analysis of the phenotypic data and expression levels was performed using two-tailed Student’s *t*-test in Excel (^∗^*P* < 0.05, ^∗∗^*P* < 0.01).

## Results

### Abscisic Acid Induced Transcription Repressors in Soybean

**FIGURE 1:**
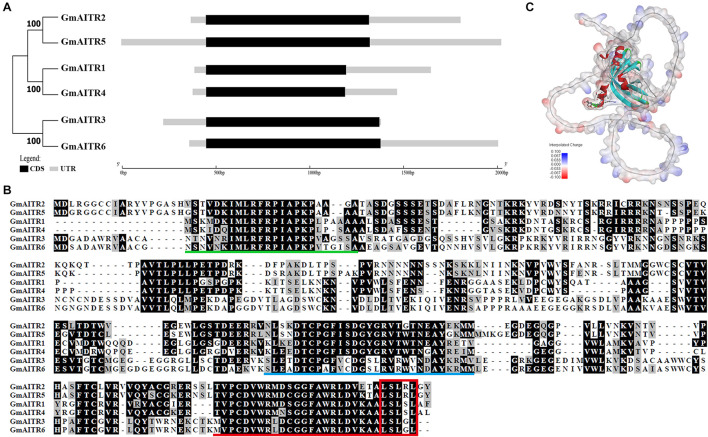
Abscisic acid (ABA) induced transcription repressors (AITRs) in Soybean. **(A)** Phylogenetic relationship and gene structures of *GmAITRs*. Coding sequence (CDS) and untranslated region (UTR) sequences were indicated in black and gray boxes, respectively, and the lengths were drawn to scale. The phylogenetic analysis was performed by using MEGA 7.0. **(B)** Amino acid sequence alignment of GmAITRs. Full-length amino acid sequences of GmAITRs were obtained from phytozome (https://phytozome-next.jgi.doe.gov/), and used for alignment on BioEdit. Identical and similar amino acids are shaded in black and gray, respectively. Conserved motifs are predicted by MEME analysis (http://meme-suite.org), and indicated by underlines in different colors. Red box indicates the LxLxL transcriptional repression motif. **(C)** Three-dimensional structure of GmAITR2 predicted by AlphaFold v2.0 (https://www.alphafold.ebi.ac.uk/). The LxLxL repression motif was highlighted in scaled-atom form and the atom charge was added as the surface of the protein.

We have previously identified that there are six genes in soybean encoding AITRs, a number identical to that in Arabidopsis (Tian et al., 2017). Similar to the Arabidopsis *AITR* genes (Tian et al., 2017), all the 6 *GmAITRs* are genes with a single exon (Figure 1A). Phylogenetic analysis shows that GmAITR2 is closely related to GmAITR5, whereas GmAITR1 is closely related to GmAITR4, and together, these four GmAITRs formed one clade. On the other hand, GmAITR3 is closely related to GmAITR6, and they formed another clade (Figure 1A). Expanded phylogenetic analysis with AITRs from the dicot plant Arabidopsis, soybean and Medicago and the monocot plant rice (*Oryza sativa*) shows that the two OsAITRs formed a distinct clade, whereas two other clades were formed by AITRs from the three dicot plants, and both of the clades contain AITRs from all the three dicot plants (Supplementary Figure 1).

Sequence alignment shows that GmAITRs shared high amino acid identity and similarity, and contain a conserved LxLxL motif at their C-terminal (Figure 1B). Protein domain assays indicates that these three conserved domains in all the GmAITRs, one at the N-terminal, one in the middle region and the third is the LxLxL motif containing domain at the C-terminal (Figure 1B and Supplementary Figure 2). In addition, GmAITRs are hydrophilic^6^ and non-transmembrane proteins^7^, and protein structure prediction with AlphaFold v2.0 (Jumper et al., 2021) indicate that all the GmAITRs have similar three-dimensional structures (Figure 1C and Supplementary Figure 3). These results suggest that GmAITRs may have similar functions.

### Expression of *GmAITRs* Is Induced by Abscisic Acid and Salt, and *GmAITRs* Function as Transcription Repressors

**FIGURE 2:**
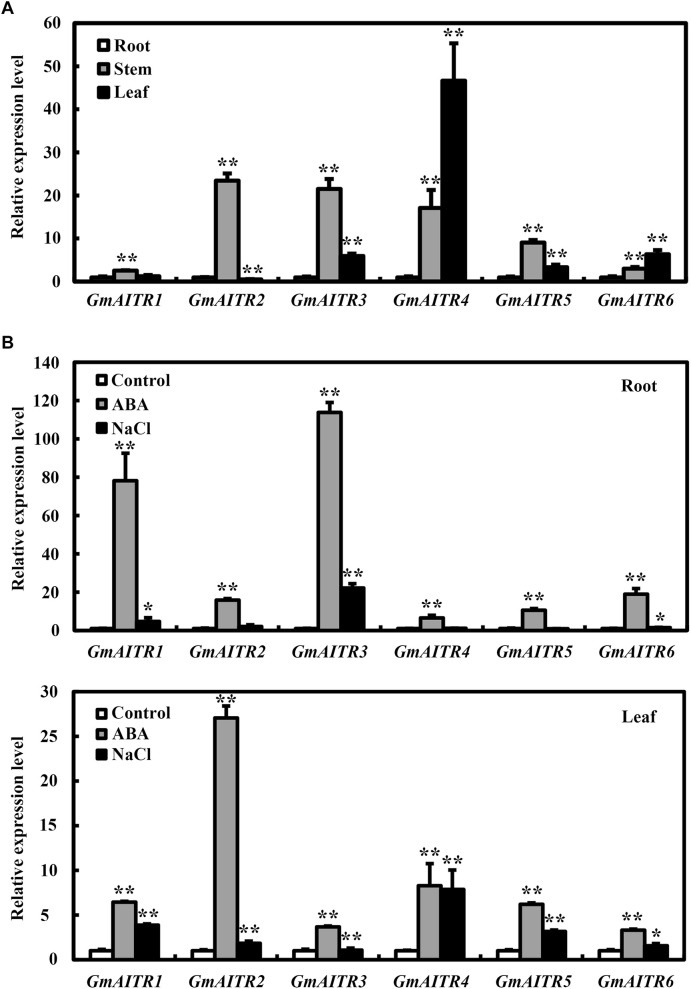
Expression of *GmAITRs* in different tissues and organs, and in response to ABA and salt treatments. **(A)** Expression of *GmAITRs* in different tissues and organs. Roots, stems and leaves were collected from 28-day-old soil-grown plants with the trifoliate leaf fully opened, total RNA was isolated and qRT-PCR was used to examine the expression of *GmAITRs*. The expression of *GmEF-1*α was used as an inner control. The expression levels of *GmAITRs* in roots were set as 1. Data represent the mean ± SD of three replicates. **(B)** Expression of GmAITRs in response to ABA and salt treatments in roots (*up panel*) and leaves (*low panel*). Fourteen-day-old seedlings grown in plastic growth bags were exposed to distilled water, 100 μM ABA or 200 mM NaCl for 6 h, then the roots and leaves were dissected, total RNA were isolated and qRT-PCR was used to examine the expression of *GmAITRs*. The expression of *GmEF-1*α was used as an inner control. The expression levels of *GmAITRs* in distilled water control were set as 1. Data represent the mean ± SD of three replicates. The experiments were repeated three times with similar results. The asterisks in the figure indicate significant different from the control (*P < 0.05; **P < 0.01).

To examine the functions of GmAITRs in ABA signaling and abiotic stress tolerance, we first examined the expression pattern of *GmAITR* genes. We found that *GmAITR* genes showed diverse expression patterns in the tissues and organs examined. In general, relative higher expression levels for all the 6 *GmAITR* genes were observed in stems, and all but *GmAITR2* also have relative higher expression levels in leaves (Figure 2A). However, difference in expression levels in different tissues and organs were observed for different *GmAITR* genes, for example, the highest expression level of *GmAITR1* was observed in stems, but it was only about 2.5-fold of that in root, whereas that of GmAITR4 in leaves was nearly 50-fold of that in root (Figure 2A).

We have previously shown that the expression of *GmAITR* genes is induced by treating excised soybean roots with ABA (Tian et al., 2017). Having shown that *GmAITRs* showed different expression patterns in the tissues and organs, we then compared ABA response of *GmAITR* genes in roots and leaves. We found that the expression levels of all the *GmAITR* genes were increased in response to ABA treatment in both root and leaves, but to different levels. For instance, an ∼80- and 110-fold increase for *GmAITR1* and *GmAITR3*, respectively in roots, and an ∼27-fold increase for *GmAITR2* in leaves (Figure 2B).

We also examined the expression of *GmAITRs* in response to salt stress, and found that salt treatment induced the expression of different *GmAITR* genes at least in roots or leaves, although to a relative lower levels when compared to ABA treatment (Figure 2B).

**FIGURE 3:**
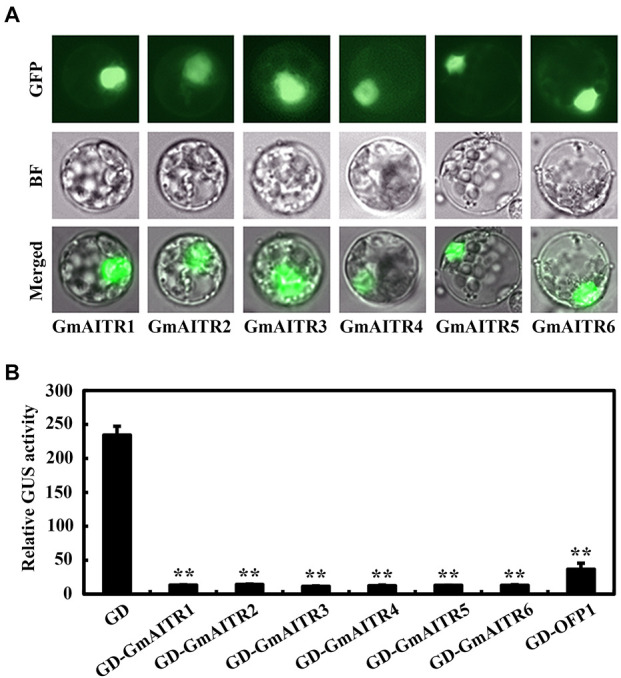
Subcellular localization and transcriptional activities of GmAITRs. **(A)** Subcellular localization of GmAITRs. Plasmid DNA of the *35S:GFP-GmAITRs* constructs was transiently transfected into protoplasts isolated from unifoliate leaves collected from 10-day-old soybean seedlings, and transfected protoplasts were incubated at room temperature and in darkness for 16–18 h. GFP florescence was visualized and pictures were taken under a florescence microscope. Up panel, GFP channel images, middle panel, bright-field (BF) images, low panel, merged images. **(B)** Transcriptional activities of GmAITRs. Plasmid DNA of the *GD-GmAITRs* constructs were co-transfected with the effector construct *LD-VP* and the reporter construct *LexA-Gal4:GUS* into protoplasts isolated from unifoliate leaves collected from 10-day-old soybean seedlings, and the transfected protoplasts were incubated at room temperature and in darkness for 22–24 h before GUS activity was assayed. Cotransfection of *GD* and *GD-AtOFP1* were used as negative and positive controls, respectively. Data represent the mean ± SD of three replicates. The experiments were repeated three times with similar results. **Significant different from the GD control (*P* < 0.01).

We further examined subcellular localization and transcriptional activity of GmAITRs in soybean protoplasts. Similar to the results observed in transfected Arabidopsis protoplasts (Tian et al., 2017), GmAITRs were localized in nucleus (Figure 3A), and they repressed the expressed *Gal4:GUS* reporter gene when recruited to the *Gal4* promoter by the fused Gal4 DNA binding domain (Figure 3B). These results suggest that GmAITRs function as transcription repressors in soybean.

### Generation of Genome Edited Transgene-Free Mutants for *GmAITR* Genes

Our previously studies have shown that AITRs are conserved in angiosperms, and *AITR* genes may be good targets for CRISPR/Cas9 genome editing to improve abiotic stress tolerance in crops (Tian et al., 2017; Chen et al., 2021; Wang et al., 2021). Our results described above indicate that GmAITRs and Arabidopsis AITRs shared similar features, we therefore decided to generate transgene-free mutants of *GmAITR* genes by using CRISPR/Cas9 genome editing, and examine their response to ABA and abiotic stresses.

Two different CRISPR/Cas9 constructs were generated by using the *pYL-CRISPR/Cas9_*ubi*_-B* vector (Ma et al., 2015), and each construct contains three target sequences with each is aimed to target a pair of *GmAITR* genes. The Wm82 wild type soybean was used for plant transformation, and gene edited status were examined in T1 plants, and transgene-free homozygous mutants were isolated from progeny of gene edited T1 plants. Editing of *GmAITR3* and *GmAITR6* were observed in T1 plants generated with one construct, and editing of *GmAITR2*-*GmAITR6* were observed in T1 plants generated with another construct. Finally, transgene-free *gmaitr3 gmaitr6* (*gmaitr36*) double and *gmaitr2 gmaitr3 gmaitr4 gmaitr5 gmaitr6* (*gmaitr23456*) quintuple homozygous mutants were obtained from construct one and two transformed plants, respectively.

**FIGURE 4:**
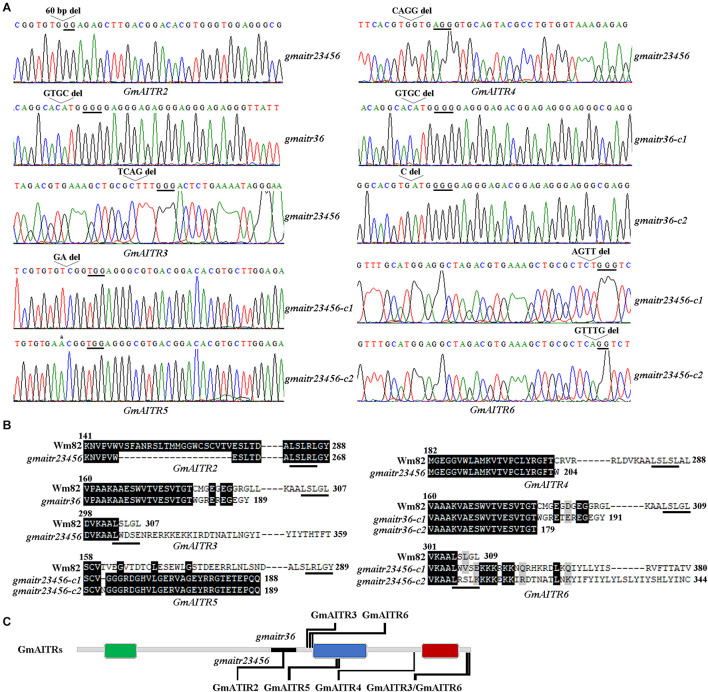
Generation of the *gmaitr36* double and the *gmaitr23456* quintuple mutants. **(A)** Editing status of *GmAITR* genes in the *gmaitr36* double and the *gmaitr23456* quintuple mutants. The mutants were generated by transforming Wm82 wild type soybean with the two CRISPR/Cas9 constructs. DNA was isolated form T1 plants and sequenced to identify gene edited mutants, transgene-free progeny of the edited T1 plants was sequenced to identify homozygous mutants. Underlines indicate the PAM sites. Open arrow heads indicate nucleotide deletions, and asterisk indicates nucleotide insertion. **(B)** Amino acid sequence alignments of GmAITRs in the Wm82 wild type and the *gmaitr36* double and the *gmaitr23456* quintuple mutant plants. Numbers above the sequences indicate the amino acid positions of corresponding GmAITRs in the Wm82 wild type plants, numbers at the end of the sequence indicate the total numbers of amino acids of the corresponding GmAITRs in the Wm82 wild type or the *gmaitrs* mutants, and underlines indicate the LxLxL transcriptional repression motifs in the corresponding GmAITRs. **(C)** Schematic diagram of the positions in GmAITRs where amino acids were altered in the *gmaitr36* double and the *gmaitr23456* quintuple mutants. Colored boxes indicate the conserved motifs of GmAITRs predicted by using MEME (http://meme-suite.org), black line in the gray line indicate the deletion of 20 amino acids of GmAITR2 in the *gmaitr23456* quintuple mutants.

In all the mutants obtained, either a single nucleotide insertion or one to up to 60 nucleotides deletion was occurred at the target sites for the *GmAITR* genes (Figure 4A), resulting in changes of amino acid sequence of the corresponding GmAITR proteins. In both *gmaitr36* double mutants, amino acid substitutions and premature stop occurred in GmAITR3, whereas amino acid substitutions and premature stop occurred in GmAITR6 in the *gmaitr36-c1* double mutant, and immediately premature stop occurred in GmAITR6 in the *gmair36-c2* double mutant, respectively (Figure 4B). In the *gmaitr23456* quintuple mutants, 20 amino acids deletion occurred in GmAITR2, an amino acid substitution and premature stop occurred in GmAITR4, and amino acid substitutions and premature stop occurred in GmAITR5 (Figure 4B). However, nucleotides deletions in *GmAITR3* and *GmAITR6* in the *gmaitr23456* quintuple mutants led to amino acid substitution and addition of extra amino acids in corresponding GmAITR proteins (Figure 4B). The positions of amino acids changes in the GmAITR proteins for the *gmaitr36* double and *gmaitr23456* quintuple mutants were diagrammed in Figure 4C. Moreover, protein structures of genome edited GmAITRs were predicted by AlphaFold v2.0 (Supplementary Figure 3), and obvious differences can be found in *gmaitr36* double mutants for protein GmAITR3 and GmAITR6. In *gmaitr23456* quintuple mutants, protein structures of GmAITR4 and GmAITR5 were severely damaged compared with wild type, while GmAITR2, GmAITR3, and GmAITR6 preserved similar structures as wild type.

### The *gmaitr* Mutants Are Hypersensitivity to Abscisic Acid

**FIGURE 5:**
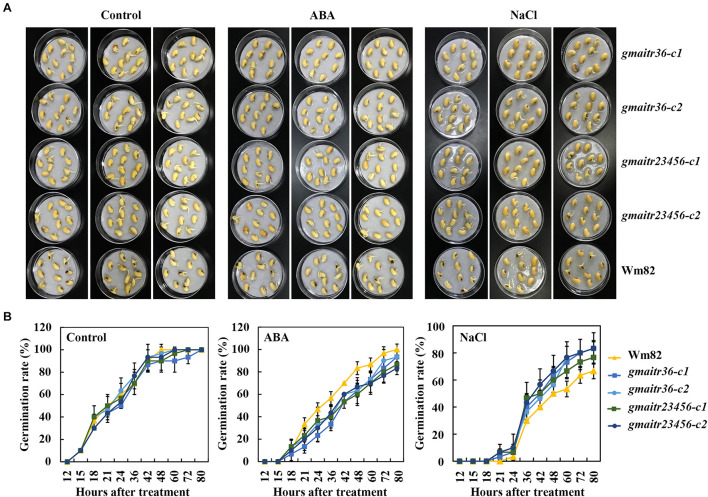
Seed germination of the Wm82 wild-type and the *gmaitr* mutants under ABA and salt treatments. **(A)** Germination of the Wm82 wild-type and the *gmaitr* mutants seeds in plates containing ABA and salt solutions. Healthy and uniform sized seeds of the Wm82 wild-type and the *gmaitr* mutants were incubated on two layers of filter papers in Petri dishes, treated with 100 μM ABA, 200 mM NaCl, or distilled water as a control. Ten seeds were from each genotype were used for the treatment and every treatment contains three replicates. The pictures were taken 48 h after treatment. **(B)** Seed germination rates the Wm82 wild-type and the *gmaitr* mutant seeds in plates containing ABA and salt solutions. Seeds germinated were counted at indicated time points and seed germination rates were calculated. Data represent the mean ± SD of three replicates. The experiments were repeated three times with similar results.

By using seed germination assays, we examined ABA response of the *gmaitr* mutants generated. Different from the results observed in the Arabidopsis *aitr* mutants, which showed a decreased ABA sensitivity (Tian et al., 2017; Chen et al., 2021), we found that seeds of all the *gmaitr* mutants were more sensitivity to ABA treatment when compared to the Wm82 wild type seeds (Figure 5A). Quantitative assays show that no difference was observed for the Wm82 wild type and the mutant seeds on control plates (plates soaked with distilled water, which is the dissolvent of ABA and salt solution), seeds of all the plants reached a maximum germinate rate, i.e., ∼100% 48 h after treatment. On the other hand, when compared to the Wm82 wild type seeds, a reduced germination rate was observed for seeds of all the mutants on the ABA treated plates (Figure 5B), indicating that ABA sensitivity in the mutants was increased. However, we found that germination rate of the *gmaitr36* double mutant seeds is largely indistinguishable from that of the *gmaitr23456* quintuple mutant seeds (Figure 5B).

**FIGURE 6:**
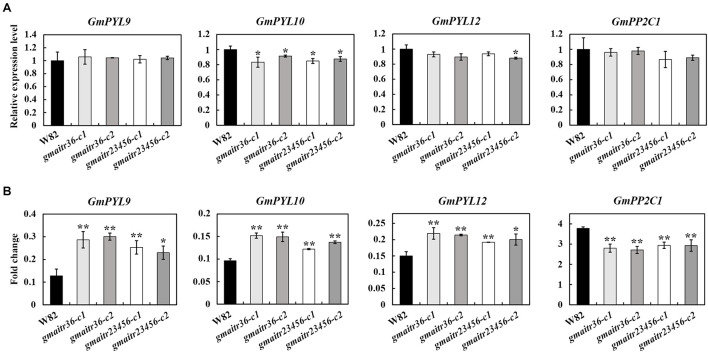
ABA response of the ABA signaling key regulator genes in the Wm82 wild type and the *gmaitr* mutant plants. **(A)** Basal expression levels of ABA signaling key regulator genes in the Wm82 wild type and the *gmaitr* mutant plants. RNA was isolated from 14-day-old seedlings grown in plastic growth bags, total RNA was isolated and qRT-PCR was used to examine the expression of ABA signaling key regulator genes. The expression of *GmEF-1*α was used as an inner control. The expression levels of the corresponding genes in the Wm82 wild type were set as 1. Data represent the mean ± SD of three replicates. **(B)** ABA responses of the ABA signaling key regulator genes in the Wm82 wild type and the *gmaitr* mutant plants. Fourteen-day-old seedlings of the Wm82 wild type and the *gmaitr* mutant plants grown in plastic growth bags were treated with 100 μM ABA or solvent as control for 6 h, roots were collected, total RNA was isolated and qRT-PCR was used to examine the expression of ABA signaling key regulator genes. The expression of *GmEF-1*α was used as an inner control. Fold changes were calculated by comparing the expression levels of the corresponding genes in ABA-treated and control seedlings. Data represent the mean ± SD of three replicates. The experiments were repeated three times with similar results. The asterisks indicate significant differences (*P < 0.05, **P < 0.01).

Our previously results indicated that AITRs in Arabidopsis function as feedback regulators in ABA signaling by inhibiting ABA responses of some ABA signaling regulators genes (Tian et al., 2017; Chen et al., 2021). Having shown that ABA response in the *gmatir* mutants was affected, we further examined if expression levels of the core ABA signaling regulator genes may be changed in the *gmaitr* mutants. We treated the *gmaitr* mutants and Wm82 seedlings with different concentration of ABA solution, and ABA key regulator genes were significantly induced in soybean seedlings treated with 100 μM ABA, thus 100 μM ABA was used for expression analysis. We found that the basal expression levels of some ABA signaling key regulator genes identified previously (Bai et al., 2013), including the GmPYL receptor genes Gm*PYL9*, Gm*PYL10*, *GmPYL12*, and the PP2C phosphatase gene *GmPP2C1* remained largely unchanged in the *gmaitr* mutants (Figure 6A). However, ABA induced responses of these genes were reduced in the *gmaitr* mutants, even though little, if any difference was observed between the *gmaitr36* double and the *gmaitr23456* quintuple mutants (Figure 6B).

### The *gmaitr* Mutant Plants Are Tolerant to Salt Stress

Changes in the expression levels of the ABA signaling regulator genes including Arabidopsis *AITR* genes have been shown to affect plant abiotic stress tolerance (Fujita et al., 2009; Park et al., 2015; Yoshida et al., 2015; Zhao et al., 2016; Tian et al., 2017; Chen et al., 2021), but so far only *aitr* mutants showed enhanced tolerance to drought and salt, make *AITRs* good candidate genes for CRISPR/Cas9 genome editing to improve abiotic stress tolerance in plants (Tian et al., 2017; Chen et al., 2021).

To examine if mutation of *GmAITR* genes may indeed improve abiotic stress tolerance in soybean, we first examined the effects of salt treatment on seed germination of the *gmaitr* mutants. We found that the *gmaitr* mutant seeds showed enhanced tolerance to salt treatment (Figure 5A), and quantitative assays showed that an increased germination rate were observed for seeds of *gmaitr* mutants at all the time points examined (Figure 5B). But similar to the results observed with ABA treatment, little, if any difference was observed between the *gmaitr36* double and the *gmaitr23456* quintuple mutant seeds (Figure 5B).

**FIGURE 7:**
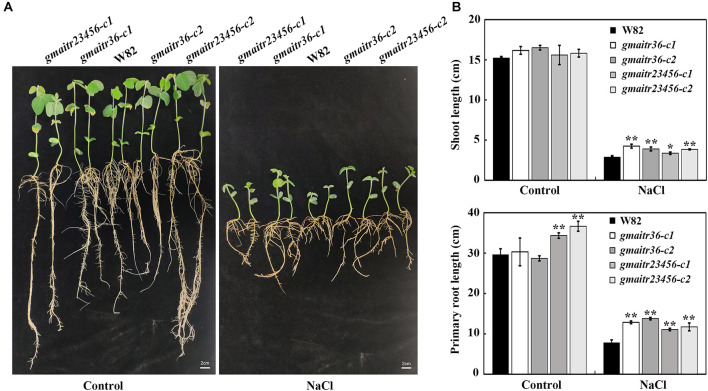
Salt tolerance of the *gmaitr* mutants. **(A)** Seedlings of the Wm82 wild type plant and the *gmaitr* mutants in response to salt treatment. Seeds were germinated and grown in distilled water-containing plastic growth bags for 3 days in a growth chamber, and then the distilled water was replaced by 200 mM NaCl or fresh distilled water as a control. For each treatment, seedlings were grown in two growth bags with two plants for each genotype. Two weeks after the treatment, representative seedlings were taken out from the growth bags and images were taken using a digital camera. Bar, 2 cm. **(B)** Shoot and primary root length of the control and salt treated seedlings of Wm82 wild type and the *gmaitr* mutant plants. Length of the shoot and primary root length of the Wm82 wild type and the *gmaitr* mutant seedlings were measured 2 weeks after the treatment. Data represent the mean ± SD of four seedlings. The asterisks indicate significant differences (*P < 0.05, **P < 0.01).

We then examined the effects of salt treatment on seedling growth of the *gmaitr* mutants. As shown in Figure 7A, the *gmaitr* mutant seedlings showed enhanced tolerance to salt treatment, as they produced longer roots and shoots when compared with the Wm82 wild type seedlings (Figure 7B).

**FIGURE 8:**
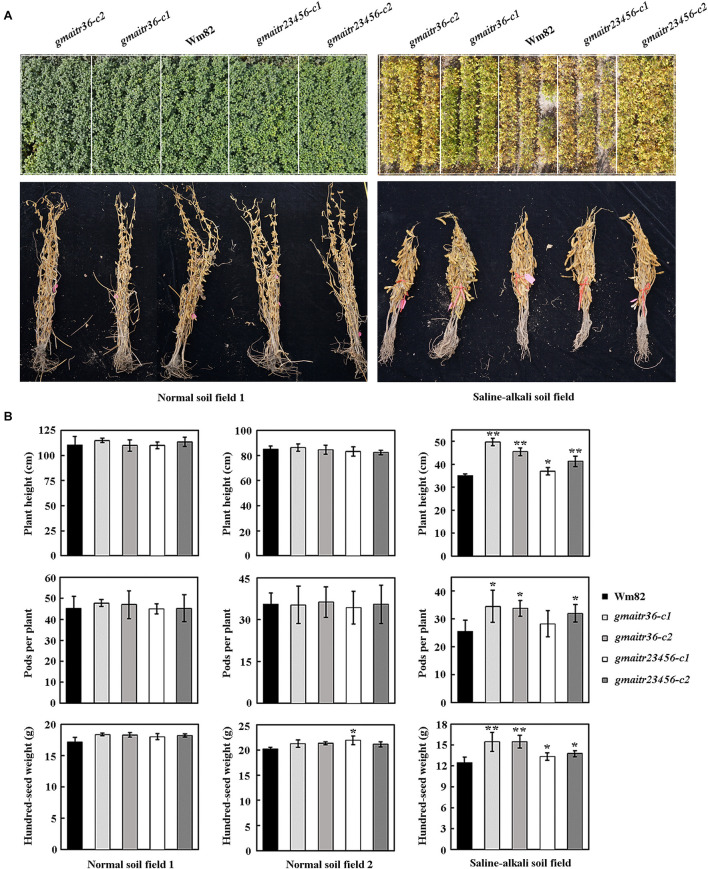
Field production of the Wm82 wild type and the *gmaitr* mutant plants in normal and saline-alkali soil lands. **(A)** Plants of the Wm82 wild type and the *gmaitr* mutants in normal and saline-alkali soil lands. The Wm82 wild-type and the *gmaitr* mutants were grown in two normal soil and one saline-alkali land (pH 8.1–9.8; soluble saline 0.1–0.7%) for field production analysis in the year 2020. Seeds were planted in plots by genotypes. Each plot in normal fields includes four rows, and plots in saline-alkali field include three rows. Numbers of seeds planted in a row for each plot were the same. *Upper panel*, field images of 4-month-old plans from one of the normal soil land the saline-alkali soil land. The white frames were used to indicate the edges of the plots. *Low panel*, images of five bundled representative mature plants for each genotype from one of the normal soil land the saline-alkali soil land. **(B)** Yield indexes of the Wm82 wild type and the *gmaitr* mutant plants in normal and saline-alkali soil lands. The Wm82 wild-type and the *gmaitr* mutant plants were harvested and plants randomly selected were used for yield indexes measurement, including plant height, pods produced per plant, and hundred-seed weight. For each field, the measurement was repeated four times with four different set of plants. Each set of plants contain five randomly selected plants from each plot. Data represent the mean ± SD of at least four replicates. The asterisks indicate significant differences (*P < 0.05; **P < 0.01).

At last, we compared growth and yield of the Wm82 wild type and the *gmaitr* mutant plants in both normal soil field and saline-alkali soil field. We found that the *gmaitr* mutant plants are morphological similar to the Wm82 wild type plants in the normal soil field, but growth better in the saline soil field (Figure 8A). Both the Wm82 wild type and the *gmaitr* mutant plants reached a height of ∼110 cm at mature stage, with ∼45 pods per plant, and produced seed with hundred-seed weight of ∼18 g (Figure 8B). Plants height, number of pods per plants and hundred-seed weight were all dramatically decreased in the saline-alkali soil field, however, the *gmaitr* mutant plants were less affected (Figure 8B).

## Discussion

Even though CRISPR/Cas9 genome editing has been successfully used to improve important agronomic traits in several different crops (Ma et al., 2015; Gao et al., 2016; Li X. et al., 2017; Lu et al., 2017; Shimatani et al., 2017; He et al., 2018; Zsögön et al., 2018; Chen et al., 2019), identification of suitable candidate genes in ABA signaling pathway for genome editing to improve abiotic stress tolerance in crops is a big challenge. In soybean, several different types of transcription factors involved in abiotic stress tolerance have been reported to be related to ABA signaling pathway, such as the AP2/ERF transcription factor GmERF3 (Zhang et al., 2009), the bZIP transcription factor GmbZIP1, GmbZIP15 and GmFDL19 (Gao et al., 2011; Li Y. et al., 2017; Zhang et al., 2020), the R2R3 MYB transcription factor GmMYB84 (Wang et al., 2017), the WRKY transcription factor GmWRKY12 and GmWRKY54 (Shi et al., 2018; Wei et al., 2019), and the NAC transcription factor GmSIN1, GmNAC06 and GmNAC8 (Li et al., 2019, 2021; Yang et al., 2020). However, among all these transcription factors, only GmbZIP15 functioned as a negative regulator of abiotic stress tolerance in soybean, yet no enhanced tolerance was observed in the transgenic soybean plants expressing a repressor form of GmbZIP15 (Zhang et al., 2020). These results suggest that none of these transcription factor genes can serve as targets for CRISPR/Cas9 gene editing to improve abiotic stress tolerance in soybean.

We have previously identified AITRs as a novel family of transcription factors conserved in angiosperms, and loss-of-function of *AITR* genes enhanced abiotic stress tolerance in Arabidopsis without fitness costs, indicating that AITRs may be good candidates for gene editing to improve abiotic stress tolerance in crops (Tian et al., 2017; Chen et al., 2021). By using of a combination of different assays including gene expression assays, transcriptional activity assays, generation of transgene-free gene edited mutants, and physiological and field yield analysis, we show that GmAITRs are ABA and salt inducible transcription repressors, and *GmAITRs* can be targeted to improve salinity stress tolerance in soybean.

First, we show that the expression of *GmAITRs* was induced by both ABA and salt treatments, eventhough these genes have different expression pattern, and there are difference among these genes in responses to ABA and salt (Figure 2). Second, we found that, similar to the results observed in Arabidopsis protoplasts (Tian et al., 2017), GmAITR proteins localized in nucleus and they repressed reporter gene expression in soybean protoplasts (Figure 3). Third, ABA inhibited seed germination was affected in the *gmaitr* mutants (Figure 5), and ABA response of some ABA signaling key regulator genes was altered in the *gmaitr* mutants (Figure 6). These results suggest that GmAITRs are ABA responsive transcription repressors and they regulate ABA response in soybean via affecting ABA signaling. Forth, the *gmaitr* mutants showed enhanced tolerance to salt in both seed germination and seedling growth assays (Figures 5, 7). Last but not least, field experiments suggest that the *gmaitr* mutants performed better in the saline-alkali soils when compared to the Wm82 wild type plants (Figure 8). These results suggest that genome editing of GmAITR genes is able to enhance salt tolerance in soybean.

It should be noted that in ABA inhibited seed germination assays, the *gmaitr* mutants showed increased sensitivity to ABA (Figure 5), a result different from that of the Arabidopsis *aitr* mutants, which showed decreased sensitivity to ABA (Tian et al., 2017; Chen et al., 2021), suggest that there is some difference between GmAITRs and Arabidopsis AITRs in regulating ABA responses. However, the *gmaitr* mutants also showed enhanced tolerance to salt (Figures 5, 7), similar to that observed in the Arabidopsis *aitr* mutants (Tian et al., 2017; Chen et al., 2021), making them good targets for genome editing to improve abiotic stress tolerance in soybean.

We also noted that the *gmaitr23456* quintuple mutants are largely indistinguishable to the *gmaitr36* double mutants in both ABA and salt tolerance assays, and in field growth conditions (Figures 5, 7, 8). Even though we cannot rule out the possibility that some of the *GmAITRs* may have a dominate roles in regulation ABA response and salt tolerance, as we previously observed for the Arabidopsis *AITRs* (Chen et al., 2021). Based on the conserved motif analysis (Figure 4) and protein structure prediction results (Supplementary Figure 3), a possible explanation is that the editing to *GmAITR2*, *GmAITR3*, and *GmAITR6* in the *gmaitr23456* quintuple mutants may not led to loss-of-function of these genes. First, as the genome editing of *GmAITR2* in the *gmaitr23456* quintuple mutants only resulted in a deletion of 20 amino acids outside the conserved motifs (Figure 4), whereas genome editing of both *GmAITR3* and *GmAITR6* in the *gmaitr23456* quintuple mutants only disrupted the LxLxL motif at the C-terminal of GmAITR3 and GmAITR6, respectively. Our previously results with Arabidopsis AITRs have already shown that the deletion of LxLxL motif affected AITRs’ transcriptional repression activities, but they are still able to function as transcription repressors (Tian et al., 2017). Second, according to the three-dimensional protein structure prediction, the protein binding pockets structure, which is important for protein functionality (Stank et al., 2016), were barely not damaged for GmAITR2, GmAITR3 and GmAITR6 in the *gmaitr23456* quintuple mutants compared with wild type (Supplementary Figure 3). Therefore, it will be of great interest to generate high-order loss-of-function mutants of *GmAITR* genes and to examine if increased tolerance to abiotic stresses can be achieved, and if there are any fitness costs. It will be of great interest to compare physiological/biochemical index in the Wm82 and the *gmaitr* mutants, and use more negative controls for the ABA and salt related response analysis, therefore to understand the subtle changes and physiological mechanism of GmAITR in abiotic stress tolerance. It will be also of great interest to edit *GmAITR* genes in soybean cultivars with other good agronomic traits to see if enhanced abiotic stress tolerance can be obtained without affecting these agronomic traits, thereby accelerating the molecular breeding process of soybean with different benefit agronomic traits.

On the other hand, considering that in all the major crops, AITRs are encoded by multiple genes (Tian et al., 2017), loss-of-function of a few *AITR* genes can already led to enhanced abiotic stress tolerance making it more practicable for editing *AITR* genes to improve abiotic stress tolerance in crops. After all, it is not easy to edit all the *AITR* genes simultaneously in a crop.

Nevertheless, our results show that GmAITRs are involved in the regulation of ABA response and abiotic stress tolerance in soybean, and CRISPR/Cas9 genome editing of *GmAITR* genes is able to enhance salt tolerance in soybean.

## Data Availability Statement

The original contributions presented in the study are included in the article/Supplementary Material, further inquiries can be directed to the corresponding author.

## Author Contributions

SW, BL, YD, XF, and JP conceived the study. TW and SW designed the experiments, analyzed the data, and drafted the manuscript. HT made the CRISPR/Cas9 constructs. HX and XQ generated the mutants. TW, WW, and GL examined the gene editing status in the mutants. TW, XD, SH, YL, YC, CW, and RL did the experiments. QD performed the bioinformatics analysis. All authors participated in the revision of the manuscript, read and approved the final manuscript.

## Conflict of Interest

The authors declare that the research was conducted in the absence of any commercial or financial relationships that could be construed as a potential conflict of interest.

## Publisher’s Note

All claims expressed in this article are solely those of the authors and do not necessarily represent those of their affiliated organizations, or those of the publisher, the editors and the reviewers. Any product that may be evaluated in this article, or claim that may be made by its manufacturer, is not guaranteed or endorsed by the publisher.

## Footnotes

1
http://meme-suite.org

2
https://www.alphafold.ebi.ac.uk/

3
https://www.3ds.com/

4
https://mafft.cbrc.jp/alignment/server/

5
http://skl.scau.edu.cn/home/

6
http://www.detaibio.com/sms2/protein_gravy.html

7
http://www.cbs.dtu.dk/services/TMHMM/

## References

[B1] Bai G. Yang D. Zhao Y. Ha S. Yang F. Ma J. (2013). Interactions between soybean ABA receptors and type 2C protein phosphatases. *Plant Mol. Biol.* 83 651–664. 10.1007/s11103-013-0114-4 23934343 PMC3834219

[B2] Bailey T. L. Boden M. Buske F. A. Frith M. Grant C. E. Clementi L. (2009). MEME SUITE: tools for motif discovery and searching. *Nucleic Acids Res.* 37 W202–W208. 19458158 10.1093/nar/gkp335 PMC2703892

[B3] Chen K. Wang Y. Zhang R. Zhang H. Gao C. (2019). CRISPR/Cas genome editing and precision plant breeding in agriculture. *Annu. Rev. Plant Biol.* 70 667–697. 30835493 10.1146/annurev-arplant-050718-100049

[B4] Chen S. Zhang N. Zhou G. Hussain S. Ahmed S. Tian H. (2021). Knockout of the entire family of AITR genes in *Arabidopsis* leads to enhanced drought and salinity tolerance without fitness costs. *BMC Plant Boil.* 21:137. 10.1186/s12870-021-02907-9 33726681 PMC7967987

[B5] Deshmukh R. Sonah H. Patil G. Chen W. Prince S. Mutava R. (2014). Integrating omic approaches for abiotic stress tolerance in soybean. *Front. Plant Sci.* 5:244. 10.3389/fpls.2014.00244 24917870 PMC4042060

[B6] Dong T. Park Y. Hwang I. (2015). Abscisic acid: biosynthesis, inactivation, homoeostasis and signalling. *Essays Biochem.* 58 29–48. 10.1042/bse0580029 26374885

[B7] Edwards K. Johnstone C. Thompson C. (1991). A simple and rapid method for the preparation of plant genomic DNA for PCR analysis. *Nucleic Acids Res.* 19:1349. 10.1093/nar/19.6.1349 PMC333874 2030957

[B8] Fujii H. Verslues P. E. Zhu J. K. (2007). Identification of two protein kinases required for abscisic acid regulation of seed germination, root growth, and gene expression in *Arabidopsis*. *Plant Cell* 19 485–494. 10.1105/tpc.106.048538 17307925 PMC1867333

[B9] Fujii H. Zhu J. K. (2009). *Arabidopsis* mutant deficient in 3 abscisic acid-activated protein kinases reveals critical roles in growth, reproduction, and stress. *Proc. Natl. Acad. Sci. U. S. A.* 106 8380–8385. 10.1073/pnas.0903144106 19420218 PMC2688869

[B10] Fujita M. Fujita Y. Noutoshi Y. Takahashi F. Narusaka Y. Yamaguchi-Shinozaki K. (2006). Crosstalk between abiotic and biotic stress responses: a current view from the points of convergence in the stress signaling networks. *Curr. Opin. Plant Biol.* 9 436–442. 10.1016/j.pbi.2006.05.014 16759898

[B11] Fujita Y. Nakashima K. Yoshida T. Katagiri T. Kidokoro S. Kanamori N. (2009). Three SnRK2 protein kinases are the main positive regulators of abscisic acid signaling in response to water stress in *Arabidopsis*. *Plant Cell Physiol.* 50 2123–2132. 10.1093/pcp/pcp147 19880399

[B12] Gao S. Q. Chen M. Xu Z. S. Zhao C. P. Li L. Xu H. J. (2011). The soybean GmbZIP1 transcription factor enhances multiple abiotic stress tolerances in transgenic plants. *Plant Mol.Biol.* 75 537–553. 10.1007/s11103-011-9738-4 21331631

[B13] Gao X. Chen J. Dai X. Zhang D. Zhao Y. (2016). An effective strategy for reliably isolating heritable and Cas9-free *Arabidopsis* mutants generated by RISPR/Cas9-mediated genome editing. *Plant Physiol.* 171 1794–1800. 10.1104/pp.16.00663 27208253 PMC4936589

[B14] Gosti F. Beaudoin N. Serizet C. Webb A. A. Vartanian N. Giraudat J. (1999). ABI1 protein phosphatase 2C is a negative regulator of abscisic acid signaling. *Plant Cell* 11 1897–1910. 10.2307/3871085 10521520 PMC144098

[B15] Guo J. Yang X. Weston D. J. Chen J. G. (2011). Abscisic acid receptors: past, present and future. *J. Integr. Plant Biol.* 53 469–479. 10.1111/j.1744-7909.2011.01044.x 21554537

[B16] He Y. Zhu M. Wang L. Wu J. Wang Q. Wang R. (2018). Programmed self-elimination of the CRISPR/Cas9 construct greatly accelerates the isolation of edited and transgene-free rice plants. *Mol. Plant* 11 1210–1213. 10.1016/j.molp.2018.05.005 29857174

[B17] Jumper J. Evans R. Pritzel A. Green T. Figurnov M. Ronneberger O. (2021). Highly accurate protein structure prediction with AlphaFold. *Nature* 596 583–589. 34265844 10.1038/s41586-021-03819-2 PMC8371605

[B18] Kan G. Zhang W. Yang W. Ma D. Zhang D. Hao D. (2015). Association mapping of soybean seed germination under salt stress. *Mol. Genet. Genomics* 290 2147–2162. 10.1007/s00438-015-1066-y 26001372

[B19] Katoh K. Standley D. M. (2013). MAFFT multiple sequence alignment software version 7: improvements in performance and usability. *Mol. Biol. Evol.* 30, 772–780. 10.1093/molbev/mst010 23329690 PMC3603318

[B20] Kumar S. Stecher G. Tamura K. (2016). MEGA7: Molecular Evolutionary Genetics Analysis Version 7.0 for Bigger Datasets. *Mol. Biol. Evol.* 33 1870–1874. 10.1093/molbev/msw054 27004904 PMC8210823

[B21] Li J. F. Norville J. E. Aach J. McCormack M. Zhang D. Bush J. (2013). Multiplex and homologous recombination-mediated genome editing in *Arabidopsis* and Nicotiana benthamiana using guide RNA and Cas9. *Nat. Biotechnol.* 31 688–691. 10.1038/nbt.2654 23929339 PMC4078740

[B22] Li M. Chen R. Jiang Q. Sun X. Zhang H. Hu Z. (2021). GmNAC06, a NAC domain transcription factor enhances salt stress tolerance in soybean. *Plant Mol. Biol.* 105 333–345. 10.1007/s11103-020-01091-y 33155154 PMC7858558

[B23] Li S. Wang N. Ji D. Zhang W. Wang Y. Yu Y. (2019). A GmSIN1/GmNCED3s/GmRbohBs feed-forward loop acts as a signal amplifier that regulates root growth in soybean exposed to salt stress. *Plant Cell* 31 2107–2130. 10.1105/tpc.18.00662 31227558 PMC6751118

[B24] Li X. Xie Y. Zhu Q. Liu Y. G. (2017). Targeted genome editing in genes and cis-regulatory regions improves qualitative and quantitative traits in crops. *Mol. Plant* 10 1368–1370. 10.1016/j.molp.2017.10.009 29079543

[B25] Li Y. Chen Q. Nan H. Li X. Lu S. Zhao X. (2017). Overexpression of GmFDL19 enhances tolerance to drought and salt stresses in soybean. *PLoS One* 12:e0179554. 10.1371/journal.pone.0179554 28640834 PMC5480881

[B26] Lu H. P. Liu S. M. Xu S. L. Chen W. Y. Zhou X. Tan Y. Y. (2017). CRISPR-S: an active interference element for a rapid and inexpensive selection of genome-edited, transgene-free rice plants. *Plant Biotechnol. J.* 15 1371–1373. 10.1111/pbi.12788 28688132 PMC5633759

[B27] Ma X. Zhang Q. Zhu Q. Liu W. Chen Y. Qiu R. (2015). A robust CRISPR/Cas9 system for convenient, high-efficiency multiplex genome editing in monocot and dicot plants. *Mol. Plant* 8 1274–1284. 10.1016/j.molp.2015.04.007 25917172

[B28] Manavalan L. P. Guttikonda S. K. Tran L. S. Nguyen H. T. (2009). Physiological and molecular approaches to improve drought resistance in soybean. *Plant Cell Physiol.* 50 1260–1276. 10.1093/pcp/pcp082 19546148

[B29] Matres J. M. Hilscher J. Datta A. Armario-Nájera V. Baysal C. He W. (2021). Genome editing in cereal crops: an overview. *Transgenic Res.* 30 461–498. 10.1007/s11248-021-00259-6 34263445 PMC8316241

[B30] Nekrasov V. Staskawicz B. Weigel D. Jones J. D. Kamoun S. (2013). Targeted mutagenesis in the model plant Nicotiana benthamiana using Cas9 RNA-guided endonuclease. *Nat. Biotechnol.* 31 691–693. 10.1038/nbt.2655 23929340

[B31] Park S. Y. Peterson F. C. Mosquna A. Yao J. Volkman B. F. Cutler S. R. (2015). Agrochemical control of plant water use using engineered abscisic acid receptors. *Nature* 520 545–548. 10.1038/nature14123 25652827

[B32] Paz M. Shou H. Guo Z. Zhang Z. Banerjee A. Wang K. (2004). Assessment of conditions affecting Agrobacterium-mediated soybean transformation using the cotyledonary node explant. *Euphytica* 136 167–179. 10.1023/b:euph.0000030670.36730.a4

[B33] Ray D. K. Mueller N. D. West P. C. Foley J. A. (2013). Yield trends are insufficient to double global crop production by 2050. *PLoS One* 8:e66428. 10.1371/journal.pone.0066428 23840465 PMC3686737

[B34] Rodriguez P. L. Leube M. P. Grill E. (1998). Molecular cloning in *Arabidopsis thaliana* of a new protein phosphatase 2C (PP2C) with homology to ABI1 and ABI2. *Plant Mol. Biol.* 38 879–883. 10.1023/a:1006012218704 9862504

[B35] Rushton D. L. Tripathi P. Rabara R. C. Lin J. Ringler P. Boken A. K. (2012). WRKY transcription factors: key components in abscisic acid signalling. *Plant Biotechnol. J.* 10 2–11. 10.1111/j.1467-7652.2011.00634.x 21696534

[B36] Shan Q. Wang Y. Li J. Zhang Y. Chen K. Liang Z. (2013). Targeted genome modification of crop plants using a CRISPR-Cas system. *Nat. Biotechnol.* 31 686–688. 10.1038/nbt.2650 23929338

[B37] Shi W. Y. Du Y. T. Ma J. Min D. H. Jin L. G. Chen J. (2018). The WRKY Transcription factor GmWRKY12 confers drought and salt tolerance in Soybean. *Int. J. Mol. Sci.* 19:4087. 10.3390/ijms19124087 30562982 PMC6320995

[B38] Shimatani Z. Kashojiya S. Takayama M. Terada R. Arazoe T. Ishii H. (2017). Targeted base editing in rice and tomato using a CRISPR-Cas9 cytidine deaminase fusion. *Nat. Biotechnol.* 35 441–443. 10.1038/nbt.3833 28346401

[B39] Song L. Huang S. C. Wise A. Castanon R. Nery J. R. Chen H. (2016). A transcription factor hierarchy defines an environmental stress response network. *Science* 354:aag1550. 10.1126/science.aag1550 27811239 PMC5217750

[B40] Stank A. Kokh D. B. Fuller J. C. Wade R. C. (2016). Protein binding pocket dynamics. *Acc. Chem. Res.* 49 809–815. 27110726 10.1021/acs.accounts.5b00516

[B41] Tian H. Chen S. Yang W. Wang T. Zheng K. Wang Y. (2017). A novel family of transcription factors conserved in angiosperms is required for ABA signalling. *Plant Cell Environ.* 40 2958–2971. 10.1111/pce.13058 28857190

[B42] Tiwari S. B. Hagen G. Guilfoyle T. J. (2004). Aux/IAA proteins contain a potent transcriptional repression domain. *Plant Cell* 16 533–543. 10.1105/tpc.017384 14742873 PMC341922

[B43] Umezawa T. Nakashima K. Miyakawa T. Kuromori T. Tanokura M. Shinozaki K. (2010). Molecular basis of the core regulatory network in ABA responses: sensing, signaling and transport. *Plant Cell Physiol.* 51 1821–1839. 10.1093/pcp/pcq156 20980270 PMC2978318

[B44] Vanlliyodan B. Ye H. Song L. Murphy M. Shannon J. G. Nguyen H. T. (2017). Genetic diversity and genomic strategies for improving drought and waterlogging tolerance in soybeans. *J. Exp. Bot.* 68 1835–1849. 10.1093/jxb/erw433 27927997

[B45] Wang N. Zhang W. Qin M. Li S. Qiao M. Liu Z. (2017). Drought tolerance conferred in soybean (*Glycine max*. L) by GmMYB84, a Novel R2R3-MYB transcription factor. *Plant Cell Physiol.* 58 1764–1776. 10.1093/pcp/pcx111 29016915

[B46] Wang S. Tiwari S. B. Hagen G. Guilfoyle T. J. (2005). AUXIN RESPONSE FACTOR7 restores the expression of auxin-responsive genes in mutant *Arabidopsis* leaf mesophyll protoplasts. *Plant Cell* 17 1979–1993. 10.1105/tpc.105.031096 15923351 PMC1167546

[B47] Wang T. Dong Q. Wang W. Chen S. Cheng Y. Tian H. (2021). Evolution of AITR family genes in cotton and their functions in abiotic stress tolerance. *Plant Biol.* 23 58–68. 10.1111/plb.13218 33202099

[B48] Wang W. Vinocur B. Altman A. (2003). Plant responses to drought, salinity and extreme temperatures: towards genetic engineering for stress tolerance. *Planta* 218 1–14. 10.1007/s00425-003-1105-5 14513379

[B49] Wei W. Liang D. W. Bian X. H. Shen M. Xiao J. H. Zhang W. K. (2019). GmWRKY54 improves drought tolerance through activating genes in abscisic acid and Ca^2+^ signaling pathways in transgenic soybean. *Plant J.* 100 384–398. 10.1111/tpj.14449 31271689

[B50] Xiong L. Li C. Li H. Lyu X. Zhao T. Liu J. (2019). A transient expression system in soybean mesophyll protoplasts reveals the formation of cytoplasmic GmCRY1 photobody-like structures. *Sci. China Life Sci.* 62 1070–1077. 10.1007/s11427-018-9496-5 30929191

[B51] Xu Y. Lu Y. Xie C. Gao S. Wan J. Prasanna B. M. (2012). Whole-genome strategies for marker-assisted plant breeding. *Mol. Breeding* 29 833–854. 10.1007/s11032-012-9699-6

[B52] Yang C. Huang Y. Lv W. Zhang Y. Bhat J. A. Kong J. (2020). GmNAC8 acts as a positive regulator in soybean drought stress. *Plant Sci.* 293:110442. 10.1016/j.plantsci.2020.110442 32081255

[B53] Yoshida T. Fujita Y. Maruyama K. Mogami J. Todaka D. Shinozaki K. (2015). Four *Arabidopsis* AREB/ABF transcription factors function predominantly in gene expression downstream of SnRK2 kinases in abscisic acid signaling in response to osmotic stress. *Plant Cell Environ.* 38 35–49. 10.1111/pce.12351 24738645 PMC4302978

[B54] Yoshida T. Mogami J. Yamaguchi-Shinozaki K. (2014). ABA-dependent and ABA-independent signaling in response to osmotic stress in plants. *Curr. Opin. Plant Biol.* 21 133–139. 25104049 10.1016/j.pbi.2014.07.009

[B55] Zhang G. Chen M. Li L. Xu Z. Chen X. Guo J. (2009). Overexpression of the soybean GmERF3 gene, an AP2/ERF type transcription factor for increased tolerances to salt, drought, and diseases in transgenic tobacco. *J. Exp. Bot.* 60 3781–3796. 10.1093/jxb/erp214 19602544 PMC2736888

[B56] Zhang M. Liu Y. Cai H. Guo M. Chai M. She Z. (2020). The bZIP transcription factor GmbZIP15 negatively regulates salt- and drought-stress responses in soybean. *Int. J. Mol. Sci.* 21:7778. 10.3390/ijms21207778 33096644 PMC7589023

[B57] Zhang Y. He J. Wang Y. Xing G. Zhao J. Li Y. (2015). Establishment of a 100-seed weight quantitative trait locus-allele matrix of the germplasm population for optimal recombination design in soybean breeding programmes. *J. Exp. Bot.* 66 6311–6325. 10.1093/jxb/erv342 26163701

[B58] Zhao Y. Chan Z. Gao J. Xing L. Cao M. Yu C. (2016). ABA receptor PYL9 promotes drought resistance and leaf senescence. *Proc. Natl. Acad. Sci. U. S. A.* 113 1949–1954. 10.1073/pnas.1522840113 26831097 PMC4763734

[B59] Zsögön A. Čermák T. Naves E. R. Notini M. M. Edel K. H. Weinl S. (2018). *De novo* domestication of wild tomato using genome editing. *Nat. Biotechnol.* 36, 1211–1216. 10.1038/nbt.4272 30272678

