# Supplementary material for: Mutation of GmAITR Genes by CRISPR/Cas9 Genome Editing Results in Enhanced Salinity Stress Tolerance in Soybean
Source: Front Plant Sci. 2021 Nov 26;12:779598. doi: 10.3389/fpls.2021.779598 (PMC8660858; doi:10.3389/fpls.2021.779598)
Supplement: Supplementary file 1 [file Presentation_1.pdf]

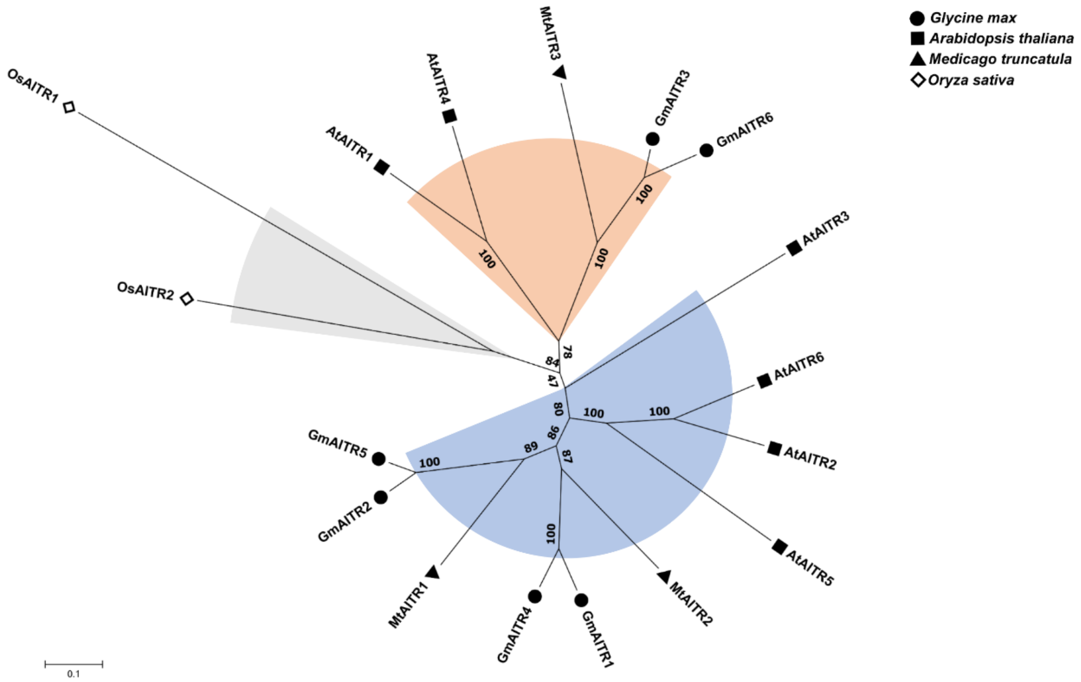

Figure S1. Phylogenetic analysis of AITR gene family in soybean, Arabidopsis, Medicago and rice.

Full-length amino acid sequences of the AITRs from soybean, Arabidopsis, Medicago and rice were obtained from phytozome (https://phytozome-next.jgi.doe.gov/), and used to generate Neighbor-joining phylogenetic tree by using MEGA 7.0. The three clades were shaded with distinct colors. The scale bar indicates 0.1 amino acid substitution per site.

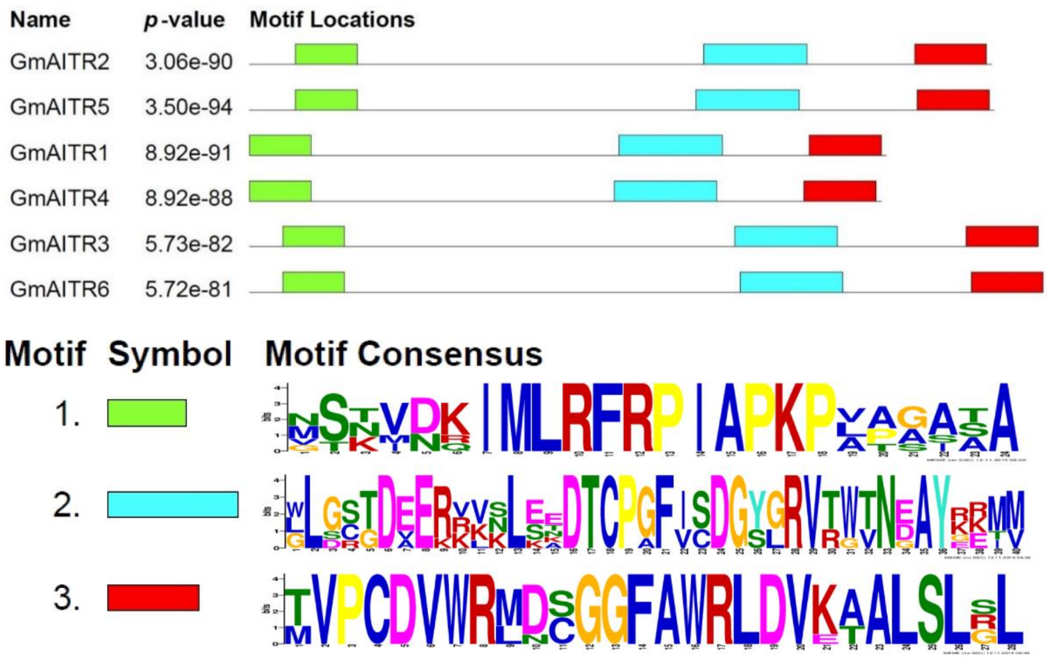

Figure S2. Conserved motif in the GmAITR proteins.

Full-length amino acid sequences of the GmAITRs were used to identify conserved motifs. Conserved motifs were identified and the detailed information of the identified motifs was analyzed by using MEME (http://meme-suite.org). The three conserved motifs identified were indicated by different colored boxes.

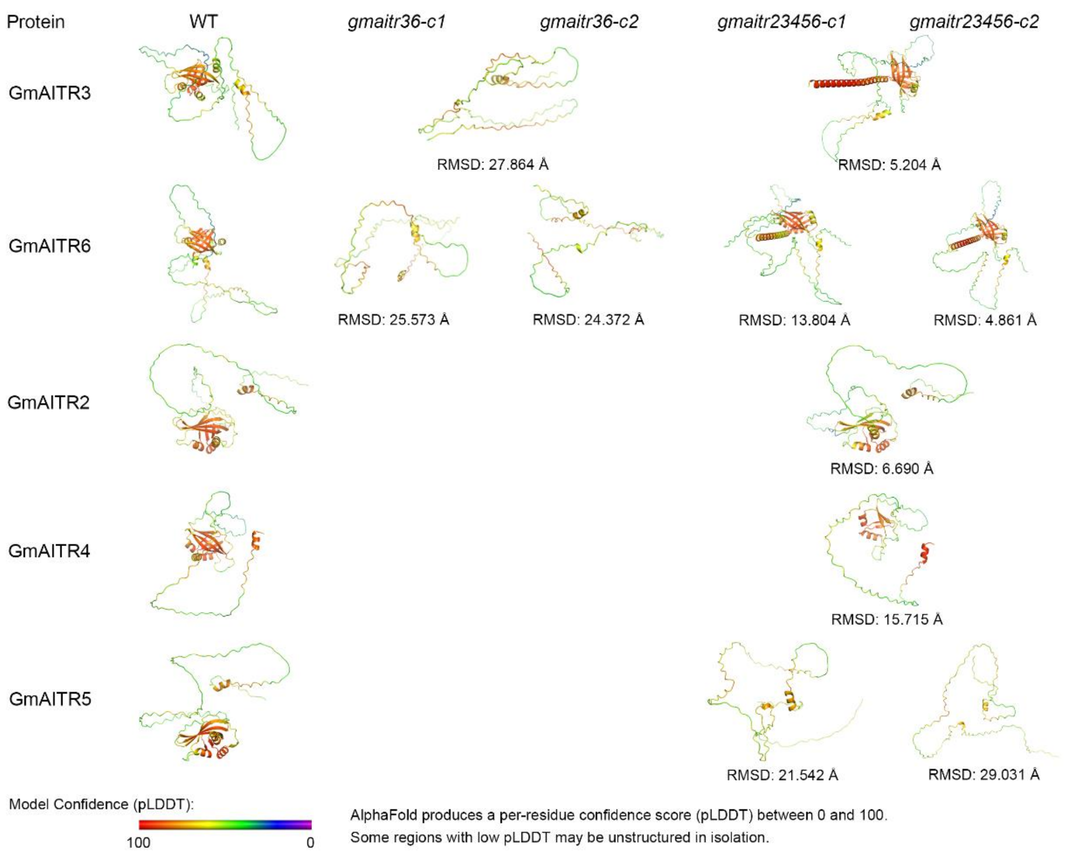

Figure S3. Predicated structures of the GmAITR proteins in the wild type and the *gmaitr* mutant plants.

Full-length amino acid sequences of the GmAITRs in the wild type and the genome edited *gmaitr* mutant plants were used for three-dimensional structures predication by using AlphaFold v2.0 (https://www.alphafold.ebi.ac.uk/). The per-residue confidence score (pLDDT) was used to color-code the residues as predicted in the bottom symbol. RMSD value below each protein structure gives the average deviation between the corresponding atoms of wild type GmAITR and mutated GmAITR proteins: the bigger the RMSD, the more different the two structures are.
