# Supplementary material for: Mutation of GmAITR Genes by CRISPR/Cas9 Genome Editing Results in Enhanced Salinity Stress Tolerance in Soybean
Source: Front Plant Sci. 2021 Nov 26;12:779598. doi: 10.3389/fpls.2021.779598 (PMC8660858; doi:10.3389/fpls.2021.779598)
Supplement: Supplementary file 2 [file Data_Sheet_1.pdf]

Table S1. Gene-specific primer pairs for qRT-PCR

**Name**	**sequence (5'-3')**
GmEF-1α-F	TGCAAAGGAGGCTGCTAACT
GmEF-1α-R	CAGCATCACCGTTCTTCAAA
GmAITR1-F	GTACTCGCAGGCGACG
GmAITR1-R	TACTCCTCCAGCACCTACTG
GmAITR2-F	CATACGAGAAGATGATGGAGG
GmAITR2-R	TTAGTAACCCAGTCTCAAG
GmAITR3-F	CGACGGAAGCCAGAGTAG
GmAITR3-R	AAGATCTGAACCCTTGGCA
GmAITR4-F	GTGCTGGTACTCTCCGGC
GmAITR4-R	CATACTCCTCCCTCTCCCATAA
GmAITR5-F	GTACGGGAAGATGATGATGAT
GmAITR5-R	CTAATAACCCAATCTCAAGCTAAGC
GmAITR6-F	CGAAGCTGGCTCCGC
GmAITR6-R	CAAGATCTGAACGCTTACCT

Table S2. Primer pairs for vector construction

Name	sequence (5'-3')
gRT-T1-F	GGTATGTACCCGGTGCATCCGTTTTAGAGCTAGAAAT
AtU6-1-T1-R	GGATGCACCGGGTACATACCCAATCACTACTTCGTCT
gRT-T3-F	GGAGGGGTTTGGGGGCGATAGTTTTAGAGCTAGAAAT
AtU6-26-T3-R	TATCGCCCCCAAACCCCTCCCAATCACTACTTCGACTC
gRT-T5-F	GCGTGACAGGCACGTGCATGGTTTTAGAGCTAGAAAT
AtU6-1-T5-R	CATGCACGTGCCTGTCACGCCAATCACTACTTCGACTC
gRT-T2-F	GTGGTGTTCGTGTGTGACGGGTTTTAGAGCTAGAAAT
AtU6-1-T2-R	CCGTCACACACGAACACCACCAATCACTACTTCGTCT
gRT-T4-F	GAGGTTTCACGTGCAGGGTGGTTTTAGAGCTAGAAAT
AtU6-26-T4-R	CACCCTGCACGTGAAACCTCCAATCACTACTTCGACTC
gRT-T6-F	GTGAAAGCTGCGCTCAGTTTGTTTTAGAGCTAGAAAT
AtU6-1-T6-R	AAACTGAGCGCAGCTTTCACCAATCACTACTTCGACTC

Table S3. Gene-specific primers for checking genome editing status

Name	sequence (5'-3')
GmAITR1-EDIT-F	ATGTCCAAGATGGACA
GmAITR1-EDIT-R	TTAGAAAGCTAAGCTTAAGC
GmAITR2-EDIT-F	ATGGATTTAAGAGGAGGCTG
GmAITR2-EDIT-R	TTAGTAACCCAGTCTCAAG
GmAITR3-EDIT-F	GAAGCCAGAGTAGCCACGTG
GmAITR3-EDIT-R	CTTTTTTTCTTTCCCTATTTTCAGAG
GmAITR4-EDIT-F	ATGTCCAAGATCGATCAAATAATG
GmAITR4-EDIT-R	TTAGAGAGCTAAGCTTAAGC
GmAITR5-EDIT-F	CCTCCCTTTCTTCATTATTAATTC
GmAITR5-EDIT-R	ACCCTCGCCCTTCATC
GmAITR6-EDIT-F	TTACTCAGAACAACCACGTGTCA
GmAITR6-EDIT-R	CCTCTATTACTCACTTACTTTCTCATATCTGAG
